# Is healthy behavior contagious: associations of social norms with physical activity and healthy eating

**DOI:** 10.1186/1479-5868-7-86

**Published:** 2010-12-07

**Authors:** Kylie Ball, Robert W Jeffery, Gavin Abbott, Sarah A McNaughton, David Crawford

**Affiliations:** 1 Centre for Physical Activity and Nutrition Research, Deakin University, Australia; 2 Division of Epidemiology and Community Health, School of Public Health, University of Minnesota, USA

## Abstract

**Background:**

Social norms are theoretically hypothesized to influence health-related behaviors such as physical activity and eating behaviors. However, empirical evidence relating social norms to these behaviors, independently of other more commonly-investigated social constructs such as social support, is scarce and findings equivocal, perhaps due to limitations in the ways in which social norms have been conceptualized and assessed. This study investigated associations between clearly-defined social norms and a range of physical activity and eating behaviors amongst women, adjusting for the effects of social support.

**Methods:**

Self-report survey data about particular physical activity (leisure-time moderate-vigorous activity; volitional walking; cycling for transport) and eating behaviors (fast food, soft drink and fruit and vegetable consumption), and social norms and support for these, were provided by 3,610 women aged 18-46 years living in socioeconomically disadvantaged neighborhoods in Victoria, Australia.

**Results:**

Results of regression analyses showed that social norms for physical activity and eating behaviors predicted these respective behaviors relatively consistently; these associations generally remained significant after adjustment for social support.

**Conclusions:**

Acknowledging the cross-sectional study design, these data confirm theoretical accounts of the importance of social norms for physical activity and eating behaviors, and suggest that this is independent from social support. Intervention strategies aimed at promoting physical activity and healthy eating could incorporate strategies aimed at modifying social norms relating to these behaviors.

## Background

The importance of social environmental influences on health-promoting behaviors such as physical activity and healthy eating has been increasingly recognized [1-3]. Perhaps the most frequently-examined and well-established social contextual correlate of physical activity and health eating behaviors is social support, including emotional, instrumental, and informational support [1-3]. However, social norms - the standards against which the appropriateness of a certain behavior is assessed - have been described as comprising amongst the least visible, yet most powerful, forms of social control over human behavior [4,5]. Relatively few studies have examined the association of both social support and social norms with physical activity or eating behaviors within the same sample, and findings on the relative importance of these two social constructs are conflicting [5,6]. For example, Emmons et al. found that social norms, but not social support, were significant predictors of physical activity in one of the samples they studied; however, neither social construct was associated with physical activity in a second independent sample.

The idea that social norms are important determinants of healthy behaviors is widely accepted and has been incorporated into a number of theories of health behavior, such as the Theory of Planned Behavior [7] and Social Cognitive Theory [8]. A perusal of the recent research literature on the topic, however, indicates considerable heterogeneity in how social norms are conceptualized and in the methods used to measure them [5,6,9-25]. One conceptual formulation is the "descriptive norm", which refers to people's beliefs about how commonly healthy behaviors are practiced in society in general or among their families and friends. A somewhat contrasting concept is the "injunctive" norm, which refers to the beliefs people have about what other people expect or encourage others to do with regard to healthy behaviors. The latter conceptualization incorporates elements of process as well as belief, and thus overlaps considerably with other more process-oriented concepts like the concepts of social support, moral norms and outcome expectancies. Within each of these normative domains there is also considerable variability in how the concept of a norm is operationalized. For example, descriptive norms have been variously assessed as the percentage of people believed to engage in a particular behavior or the extent of agreement with statements about how most people behave. Injunctive norms are variously measured as well, such as rating how much others approve of healthy behavior and encourage it, or how much they disapprove of unhealthy behavior. In both domains there is also variability in the specificity and complexity of behavioral definitions, ranging from the very specific (e.g., exercising at a criterion level of intensity for at least 30 minutes at a time at least five days a week for the last 6 months), to the very general (e.g., eating a "healthy" diet). Additionally, the reference group for defining norms is different in different studies, ranging from people in general, to people known to the respondent, to specific peer groups with which the respondent particularly identifies.

Given the conceptualization and measurement heterogeneity of the social norms concept, it is not surprising that social norms have not been as consistently related to the behavioral outcomes they presumably influence as are more consistently-defined and perhaps more proximal influences like attitudes toward healthy behaviors, intentions to engage in those behaviors, history of engaging in the behaviors or social support for the behavior. Indeed some examples of the failure of social norm measures to fail to predict healthy behavior seem likely to be due to measurement issues. In a study by Povey, for example, descriptive social norms, injunctive social norms and social support for healthy eating all failed to predict healthy eating behavior [19]. The definition of healthy behavior, however, was a composite index using multiple nutritional criteria derived from a 63-item food frequency questionnaire. Thus, the failure to find relationships between norms and behaviors may have been due to the investigators and respondents simply having different perceptions of what constitutes healthy eating. In another study by Chatzisarantis finding weak relationships between physical activity and social norms, the measure of norms asked individuals to estimate the degree to which they felt pressure from important people in their environment to engage in physical activity for at least 30 minutes per day on at least 3 days per week for the next 5 weeks, a judgment that seems likely to be beyond the ability of many people to confidently calculate [9]. In general, therefore, it may be important to ensure that the assessment of social norms provides reasonably simple but concrete health-related behaviors as the norms for consideration, rather than either overly broad or highly specific examples such as those described above.

The present investigation aimed to investigate the relationship between clearly-defined social norms and physical activity and dietary behavior by analyzing cross-sectional data from a survey of a community sample of young women from socially disadvantaged neighborhoods in Melbourne, Australia, who were participating in a broader study focusing on resilience to obesity in socioeconomically disadvantaged neighborhoods [26,27]. We examined five different questions about descriptive social norms for physical activity and three different questions about descriptive social norms for healthy eating. Analyses related the norm to physical activity and healthy eating behaviors. The principle research question was the extent to which descriptive norms correlate with health-related behaviors. Given the consistent findings in the literature of associations between social support and health-related behaviors [2,3] as well as the potential overlap between the constructs social norms and social support, the present study also examined whether associations of social norms with health-related behaviors existed after adjusting for the effects of social support, as well as for key covariates.

## Methods

### Sample

This study used baseline data provided by 3610 women aged 18-46 years who were participants in the Resilience for Eating and Activity Despite Inequality (READI) study, a cohort study of health behaviors and obesity among women and children living in socioeconomically disadvantaged neighborhoods. Ethical approval for the study was granted by the Deakin University Human Research Ethics Committee, the Victorian Department of Education and the Catholic Education Office. Participants were randomly selected using the electoral roll from 40 rural and 40 urban suburbs (neighborhoods), that were randomly selected from the most socioeconomically disadvantaged third of all suburbs across Victoria, Australia, according to the Australian Bureau of Statistics' (ABS) Socioeconomic Index for Areas [28]. For practical reasons only suburbs with more than 1200 inhabitants and within 200 km from Melbourne were included in the sampling frame. As voting is compulsory for Australian adults, the electoral roll provides a relatively complete record of population data on Australian residents aged 18 years and over.

An initial sample of 11,940 women (150 women from each of the 80 neighborhoods, or where there were fewer than 150 women living in the neighborhood, all women within the age range within that neighborhood) were mailed a baseline survey, and a total of 4,934 returned a completed survey. Excluding from the denominator those whose surveys were marked 'return to sender' (n = 861) or who were otherwise ineligible (e.g., were deceased, or were incorrectly denoted females on the electoral roll) (n = 17), this represented a response rate of 45%. Of these 4,934 women, 571 were excluded because they no longer lived in a READI suburb, nine were excluded because they were not within the desired age range (18-46 years), three were excluded because the survey was not completed by the women it was addressed to, and two subsequently requested to be withdrawn from the study. Women who were pregnant at the time of the survey (n = 210) were excluded from analyses, as were those who had incomplete data on the measures included in this analysis (n = 556). Sociodemographic characteristics of the final sample of 3610 women are presented in Table 1.

**Table 1:** Sociodemographic and weight characteristics of sample women (N = 3610)

Characteristic	n	(%)
Age	Mean: 34.5 years (SD: 8.2)	
Level of education		
Did not complete high school	791	(21.9)
Completed high school/trade certificate/diploma	1866	(51.7)
Completed tertiary education	953	(26.4)
Marital status		
Married/de facto	2357	(65.3)
Separated/divorced/widowed	301	(8.3)
Never married	952	(26.4)
Current behavior in relation to weight		
Actively doing things to gain weight	60	(1.7)
Actively doing things to avoid gaining weight	1010	(28.0)
Actively doing things to lose weight	1380	(38.2)
Not doing anything in particular for their weight	1160	(32.1)
Number of children living in home		
None	1422	(39.4)
One	648	(18.0)
Two	938	(26.0)
Three or more	602	(16.7)

### Procedure

The survey was distributed between August 2007 and January 2008. It was entirely by self-report, assessing the women's physical activity, eating behaviors and a broad range of factors thought to influence these behaviors and obesity risk. Also included in the package were an invitation letter, a consent form, a $1 lottery ticket and a teabag. A reminder protocol [29] was employed whereby letters were sent to non-responders ten days after the initial survey package was mailed. This was followed by a second reminder letter including another copy of the survey a further ten days later. The surveys were initially pilot-tested with a convenience sample of 32 women aged 18-46 years and minor modifications were made for clarity based on the feedback received.

### Measures

#### Social norms

Descriptive social norms were defined as what the respondent perceived other people in their neighborhood or whom they knew to be doing in relation to physical activity and eating. Social norms for physical activity and eating were assessed through individual items, asking respondents to indicate their agreement with a number of statements on a 5-point Likert scale with responses ranging from 'strongly disagree' to 'strongly agree'.

Two of the items were based on Mujahid et al.'s walking environment scale [30]. These were social norms for walking, measured by the item "I often see other people walking in my neighborhood", and social norms for exercising, measured by the item "I often see other people exercising (e.g., jogging, bicycling, playing sports) in my neighborhood." The remaining six items were developed specifically for use in the current study. Social norms for walking/cycling were measured by the item "Lots of women I know walk or cycle." Social norms for exercising/playing sport were measured by the item "Lots of women I know do other forms of exercise or play sport." Social norms related to doing little physical activity were measured by the item "Lots of women I know don't do much physical activity." Social norms for fast food and soft drink consumption were measured by the items "Lots of women I know... eat fast food often" or ...drink soft drink often." Social norms for healthy eating while out were measured by the item "Lots of women I know eat healthy food when they are out". The latter was focused on foods outside of the home and was intended as a more specific question than many of those used in previous studies which simply assessed 'eating healthy foods' generally.

Initial examination of the data indicated that the response options 'strongly disagree' and 'disagree' were rarely endorsed. Responses for the social norms for walking and exercising variables were collapsed into three categories: Strongly Agree, Agree, and Do not agree (consisting of Neither agree nor disagree, Disagree, and Strongly disagree). Similarly, based on their unequal distributions, responses for the remaining six social norms variables were collapsed into dichotomous categories: Agree (consisting of Strongly agree and Agree) and Do not agree (consisting of Neither agree nor disagree, Disagree, and Strongly disagree).

#### Social support

Social support measures were adapted from Sallis et al. [31]. Respondents were asked to rate how often (during the past year) members of their family: (1) did physical activity with them; (2) encouraged them to be physically active and (3) discouraged them from sitting around too much (e.g., watching too much TV). Response options for these questions were on a Likert scale from 1 (never) to 5 (very often), plus a 'not applicable' option. Responses of 'not applicable' were re-coded to 1 (never) on the assumption that this response indicated that respondents had no immediate family and so did not receive support from family, and scores were summed for the three items (Cronbach's α = 0.65) to create a total family support for physical activity measure. Respondents were also asked to rate how often they received the same three types of support from friends or work colleagues. Response options for these questions were on a Likert scale from 1 (never) to 5 (very often), and a total friend/colleague support for physical activity measure was created by summing scores for the three items (Cronbach's α = 0.74).

Family support for healthy eating was measured by asking respondents to rate how often (during the past year) members of their family: (1) ate healthy low-fat foods with them; (2) encouraged them to eat healthy low-fat foods; and (3) discouraged them from eating unhealthy foods. Response options for these questions were on a Likert scale as described above, plus a 'not applicable' option. To create a total family support for healthy eating measure, responses of 'not applicable' were re-coded to 1 for the same reason noted above and scores were summed for the three items (Cronbach's α = 0.75). Similarly, friend/colleague support for healthy eating was measured by asking respondents to rate how often they received the same three types of support from friends or work colleagues, on a Likert scale from 1 (never) to 5 (very often). A total friend/colleague support for healthy eating measure was created by summing scores for the three items (Cronbach's α = 0.86).

#### Physical activity behavior

Physical activity was assessed using the long version of the self-administered International Physical Activity Questionnaire (IPAQ-L), a well-established survey with demonstrated test-retest reliability and validity [32]. The IPAQ-L measures the total weekly time (hours and minutes/week) spent in household/yard, leisure-time, commuting and job-related activities. The present analyses used only measures of total time spent in leisure-time moderate- and vigorous-intensity physical activity, which were summed into a single variable; leisure-time walking and walking for transport, which were summed to create a 'volitional walking' variable; and cycling for transport. The continuous physical activity behavior variables were inappropriate for use as regression outcomes due to large percentages of respondents who reported engaging in zero minutes of each of the activities. For this reason, leisure-time moderate/vigorous physical activity (LTMVPA) and total volitional walking were collapsed into tertiles (low/none, medium, high). Due to more than 80% of respondents reporting zero hours of transport cycling, this variable was collapsed dichotomously (none, some).

#### Dietary intake

Three variables were used as indicators of dietary intake: fast food/pizza consumption; soft drink consumption; and fruit/vegetable consumption. These were selected due to their established association with obesity risk [33]. These variables were assessed using a Food Frequency Questionnaire (FFQ) which was based on several previously published and validated Australian questionnaires [34-36] and assessed the frequency of consumption of fast foods, soft drink, and fruit and vegetables during the previous month.

Frequency consumption of fast foods (e.g., McDonalds**^®^**, KFC) and pizza was estimated by asking two separate questions on how frequently women reported consuming fast foods and pizza during the previous month. There were nine response categories: 'never or less than once/month', '1-3 times/month', 'once/week', '2-4 times/week', '5-6 times/week', 'once/day', '2-3 times/day', '4-5 times/day', and '6 or more times/day'. Responses for these two items were converted into daily equivalents ('Never or less than once a month' = 0 serves/day, '1-3 times a month' = 0.07 serves/day', '6 or more times a day' = 6 serves/day, etc). The daily equivalent scores for fast foods and for pizza were then summed to generate a daily equivalent score for each participant. Finally, fast food daily equivalent scores were converted back into ordinal categories ('never or less than once/month', '1-3 times a month', 'once/week', '2-4 times/week', '5-6 times/week', and 'one or more times/day'. Future references to the variable 'fast food intake' refer to this ordinal combined fast food and pizza intake variable.

Frequency of soft drink consumption was assessed with a single item about how much soft drink was usually consumed each day. Response options for this item were: 'I don't drink soft drink', 'Less than 1 serve/day', '1 serve/day'; '2 serves/day'; '3 serves/day', '4-5 serves/day', '6-7 serves/day', '8-9 serves/day', and '10 or more serves/day'.

Frequency of consumption of fruit and vegetables was assessed separately by asking about the number of servings usually eaten per day. Eight response options were 'I don't eat fruit/vegetables', 'less than one serve/day', '1 serve/day', '2 serves/day', '3 serves/day', '4 serves/day', '5 serves/day', and '6 or more serves/day'. Fruit and vegetable intakes were converted to daily equivalent scores and then combined to form a single variable. Subsequently, fruit/vegetable daily equivalent scores were rounded down to the nearest number (e.g., from 1.5 to 1), and scores representing 7 or more serves per day were combined into a single category, leaving 8 ordinal categories of fruit/vegetable intake ranging from 'less than 1 serve/day' to '7 or more serves/day.'

### Data analysis

Initially, associations between physical activity and eating behaviors, and relevant norm variables were examined via ordinal logistic regression (binary logistic regression was used for the dichotomous transport cycling outcome). At the second stage of analyses, these same associations were examined, while additionally controlling for the two corresponding support variables (support for physical activity for activity outcomes, and support for healthy eating for eating outcomes). All analyses were conducted controlling for three demographic characteristics: respondents' education, marital status, and number of children. Additionally, analyses controlled for whether respondents were trying to maintain/lose/gain weight at the time they completed the survey since different weight-related goals are likely to be associated with differing levels of activity and consumption. In order to rule out potential suppressor effects due to high collinearity amongst social norms and support variables, bivariable correlations between social norms and social support variables were examined using Pearson correlations. All correlations between social norms and support variables were below r = 0.25, thus ruling out multicollinearity. Due to the sampling strategy by which participants were recruited by their neighborhood of residence, all analyses controlled for clustering by neighborhood using robust standard errors generated by the 'cluster by' command in STATA 10.

## Results

### Leisure-time moderate/vigorous physical activity

As shown in Table 2 LTMVPA was significantly associated with the social norms for exercising and for exercise/sport. Higher norms predicted more LTMVPA. LTMVPA was negatively associated with social norms for doing little physical activity at only a trend level (p < .10). When family and friend/colleague support for physical activity were controlled for, social norms for exercising and for exercise/sport both remained significant predictors of LTMVPA (Table 2). Compared with women who did not agree that they often saw other people exercising in their neighborhoods, those who agreed or strongly agreed tended to do more LTMVPA themselves. Compared with women who did not agree that lots of women they knew do other (not walking or cycling) forms of exercise or sport, those who agreed tended to do more LTMVPA.

**Table 2:** Associations between physical activity measures and social norms, with and without control for social support

Predictors of leisure-time moderate/vigorous physical activity	Step 1^a^		Step 2^b^	
	OR	95% CI	OR	95% CI
Social norms for exercising				
Neutral/disagree/strongly disagree	1		1	
Agree	1.30**	1.11, 1.51	1.22*	1.04, 1.43
Strongly agree	1.68***	1.38, 2.06	1.49***	1.20, 1.83
Social norms for exercise/sport				
Neutral/disagree/strongly disagree	1		1	
Agree/strongly agree	1.94***	1.69, 2.22	1.69***	1.47, 1.94
Social norms for doing little physical activity				
Neutral/disagree/strongly disagree	1		1	
Agree/strongly agree	0.90^	0.79, 1.01	0.93	0.83, 1.06

**Predictors of total volitional walking**	**Step 1^a^**		**Step 2^b^**	
	**OR**	**95% CI**	**OR**	**95% CI**

Social norms for walking				
Neutral/disagree/strongly disagree	1		1	
Agree	1.41***	1.18, 1.69	1.36**	1.14, 1.63
Strongly agree	1.78***	1.44, 2.20	1.68***	1.35, 2.10
Social norms for walking/cycling				
Neutral/disagree/strongly disagree	1		1	
Agree/strongly agree	1.59 (***)	1.41, 1.80	1.50***	1.33, 1.69

**Predictors of transport cycling**	**Step 1^a^**		**Step 2^b^**	
	**OR**	**95% CI**	**OR**	**95% CI**

Social norms for walking/cycling				
Neutral/disagree/strongly disagree	1		1	
Agree/strongly agree	1.28^	0.99, 1.65	1.17	0.90, 1.52

* p < .05, ** p < .005, *** p < .0005

^ p < .10 (trend level association)

^a ^outcome regressed on social norm variables individually, controlling for respondent education, marital status, number of children, and whether they were trying to maintain/lose/gain weight

^b ^outcome regressed on social norm variables individually, controlling for family support for physical activity and friend/colleague support for physical activity, plus all previous covariates (respondent education, marital status, number of children, and whether they were trying to maintain/lose/gain weight)

### Total volitional walking

Total volitional walking was significantly positively associated with social norms for walking and for walking/cycling (Table 2). When family and friend/colleague support for physical activity were controlled for, both norm variables remained significant predictors of volitional walking. Compared with women who did not agree that they often saw other people walking in their neighborhoods, those who agreed or strongly agreed tended to do more volitional walking. Compared with women who did not agree that lots of women they knew walked or cycled, those who agreed tended to do more volitional walking.

### Transport cycling

Transport cycling was associated with social norms for walking/cycling at only a trend level (Table 2). When family and friend/colleague support for physical activity were controlled for, this association became non-significant.

### Fast food intake

As shown in Table 3 fast food intake was significantly positively associated with social norms for fast food consumption. When family and friend/colleague support for healthy eating were controlled for, social norms for fast food consumption remained a significant predictor of fast food intake. Compared with women who did not agree that many women they knew ate fast food often, those who agreed reported higher intake of fast food.

**Table 3:** Associations between dietary behaviors and social norms, with and without control for social support

Predictors of fast food consumption	Step 1^a^		Step 2^b^	
	OR	95% CI	OR	95% CI
Social norms for fast food consumption				
Neutral/disagree/strongly disagree	1		1	
Agree/strongly agree	1.34***	1.17, 1.52	1.32***	1.17, 1.51

**Predictors of soft drink consumption**	**Step 1^a^**		**Step 2^b^**	
	**OR**	**95% CI**	**OR**	**95% CI**

Social norms for soft drink consumption				
Neutral/disagree/strongly disagree	1		1	
Agree/strongly agree	1.33***	1.18, 1.50	1.33***	1.17, 1.50

**Predictors of fruit and vegetable consumption**	**Step 1^a^**		**Step 2^b^**	
	**OR**	**95% CI**	**OR**	**95% CI**

Social norms for healthy eating				
Neutral/disagree/strongly disagree	1		1	
Agree/strongly agree	1.19**	1.07, 1.33	1.12^	0.99, 1.25

* p < .05, ** p < .005, *** p < .0005

^ p < .10 (trend level association)

^a ^outcome regressed on social norm variables individually, controlling for respondent education, marital status, number of children, and whether they were trying to maintain/lose/gain weight

^b ^outcome regressed on social norm variables individually, controlling for family support for healthy eating and friend/colleague support for healthy eating, plus all previous covariates (respondent education, marital status, number of children, and whether they were trying to maintain/lose/gain weight)

### Soft drink intake

Soft drink intake was significantly associated with social norms for soft drink consumption, with greater norms predicting higher intake; this association held after controlling for family and friend/colleague support for healthy eating (Table 3).

### Fruit and vegetable intake

Fruit and vegetable intake was significantly associated with social norms for healthy eating, with greater norms for healthy eating predicting higher intakes of fruits and vegetables (Table 3). The social norms variable was associated with fruit and vegetable consumption at only a trend level when family and friend/colleague support for healthy eating were controlled for.

## Discussion

Social norms comprise a common construct of several theoretical models currently widely used to predict health-related behaviors and inform the development of behavior change interventions. Despite this, social norms remain relatively inconsistently conceptualized across studies, perhaps explaining the inconsistent findings relating social norms to the key behavioral outcomes they are hypothesized to influence. The present study assessed social norms that have been termed 'descriptive', and asked about behavioral outcomes that were neither overly general (such as 'being active') nor highly specific (such as 'exercising at a criterion level of intensity for at least 30 minutes at a time at least five days a week for the last 6 months'). Results showed that all of the social norms examined showed at least a trend level of correlation with the particular behavioral outcome they were hypothesized to influence. In all cases but two, these associations were statistically significant. Further, with the exception of social norms predicting fruits and vegetable consumption, these associations remained significant after adjusting for social support for either healthy eating or for physical activity, two constructs established as consistent predictors of their respective behavioral outcomes [2,3]. Acknowledging the cross-sectional study design, this suggests that social norms may be potentially important determinants of physical activity and eating behaviors, and that this influence may be independent of the effects of the more well-established predictor, social support.

These results are not entirely consistent with those of previous studies, in which social norms have been inconsistently associated with physical activity and healthy eating [9,19]. This may be due to our efforts to conceptualize and measure social norms using items that had good face validity, were not too broad or complex, and were likely to be clear and easily interpreted by respondents. In the Povey study, for example, the researchers used a complex healthy eating index [19], and Chatzisarantis used a broader measure of physical activity (the Godin Physical Activity Questionnaire) [9]. Our results are consistent with one of the few studies to have examined the contribution of both social norms and social support to predicting physical activity [18], which found that both constructs contributed independently to predicting leisure-time physical activity.

There are several potential explanations for the associations observed in this study between social norms and physical activity and eating behaviors. For example, women who observe many others engaging in particular physical activity or eating behaviors may come to view these behaviors are 'normative' or socially desirable, and may adopt the same behaviors due either to a positive attitude about the behaviors, a shared belief in their value, and/or a strong social urge to confirm and 'fit in' to society. Alternatively, women who engage in these behaviors themselves may consequently be more likely to come into contact with women who engage in similar behaviors. Due to the cross-sectional design of the present study, this reverse direction of effects cannot be ruled out, and the influence of social norms on behaviors should be confirmed in prospective and experimental studies.

It should be noted that in this study, descriptive norms were operationalized in relation to one referent group - neighbors/people known to the participant - while social support was operationalized in relation to another group - family/work colleagues/friends. This conceptualization was considered most theoretically appropriate with regards to the contexts in which the specific behaviors might occur (for instance, eating fruit/vegetables is more likely to occur with family/work colleagues/friends than with neighbors or others). However, we cannot rule out whether the independent role of descriptive norms and social support was observed because the two constructs tapped different referent groups, rather than because the mechanism for normative influence is independent of social support. Similarly, social norms may motivate family/colleagues' social support, a possibility that was not examined within this study. Future research investigating hypothesized mediating effects between social constructs such as these is warranted.

Strengths of this study include the large sample size and the examination of social norms-behavior associations after adjustment for the more commonly-assessed construct of social support. In addition to the cross-sectional design, limitations of the study include the self-report nature of all constructs, although established measures were used where possible (e.g., the IPAQ-L to measure physical activity). Social norms were assessed using single, non-validated items only. It is also important to note that the conceptualization of social norms in this study, that is, the extent of agreement with statements about whether other people (in general, or those known to the respondent) engage in particular physical activity or eating behaviors, represents only one of several means of operationalizing this construct. Whether social norms conceptualized in other ways are independently predictive of health-related behaviors (assessed with varying levels of specificity) remains to be investigated. Further, the majority of the social norms questions in this study asked about 'other women', rather than other people generally. The alignment between the gender of participants and of the referent group in the social norms question could have resulted in stronger associations than may be observed in mixed-gender studies. In addition, there was not always precise correspondence between the behaviours assessed with the social norms, social support, and behavioural outcome indicators. Finally, this study did not assess other types of social support (e.g., instrumental or informational support), or social constructs such as social ties or social capital; nor did it attempt to investigate a comprehensive theoretical model predicting the outcome behaviors. Inclusion of more extensive measures of social context was not possible within the constraints of the present broader study, in which a large number of intrapersonal, social and physical environmental variables were examined.

## Conclusions

Acknowledging these limitations, the present results demonstrate the potential importance of social norms as a predictor of particular physical activity and eating behaviors. The potential to modify social norms as an intervention lever for promoting increased engagement in physical activity and healthy eating is worthy of further investigation. These results can help to inform or confirm conceptual models of behavior by indicating both the predictive importance of social norms relating to healthy eating and physical activity, and their independence from the more commonly operationalized social construct, social support.

## Competing interests

The authors declare that they have no competing interests.

## Authors' contributions

KB and RWJ carried out background research and drafted the manuscript. GA performed the statistical analyses and helped to draft the manuscript. KB and DC conceived the idea for and implemented the READI study, and developed the measures and methods. SAM and DC helped to draft the manuscript. All authors read and approved the final manuscript.
